# Fatty Acid Profile, Conjugated Linoleic Acid Content, and Lipid Quality Indices in Selected Yogurts Available on the Polish Market

**DOI:** 10.3390/ani12010096

**Published:** 2022-01-01

**Authors:** Beata Paszczyk, Marta Czarnowska-Kujawska

**Affiliations:** Department of Commodity and Food Analysis, The Faculty of Food Sciences, University of Warmia and Mazury in Olsztyn, 10-726 Olsztyn, Poland; paszczyk@uwm.edu.pl

**Keywords:** yogurts, bio yogurts, probiotic yogurts, eco yogurts, fatty acids, CLA, lipid quality indices

## Abstract

**Simple Summary:**

Yogurts constitute the most popular fermented milk product. They are usually produced from cow’s milk through lactic acid fermentation using *Streptococcus* *salivarius* subs. *thermophilus* and *Lactobacillus delbruecki* subs. *bulgaricus*. Yoghurt’s nutrient composition is based on the composition of the milk used for its production. There are many factors, including genetic and individual mammalian differences, feed, stage of lactation, age, and environmental factors such as season of the year, that have an impact on the final product composition and quality. Not only milk processing (temperature, exposure to light, time of heating, or storage conditions), but also the process of lactic acid fermentation and the resultant changes in milk constituents can have an effect on the nutritional value of the yoghurt. Various additives added to the yogurt can significantly improve the sensory and health related properties of milk fermented products. Finally, yogurt nutritional composition and quality is subject to changes due to the source and type of milk solids added before fermentation, species and strains of bacteria used in the process, as well as other factors, such as fermentation time and temperature. The fatty acid profile of milk and dairy products is an important factor affecting their nutritional value. The aim of this study was to determine the fatty acid composition, the content of *cis*9*trans*11 C18:2 (CLA), and lipid quality indices in yogurts available to consumers in retail sale.

**Abstract:**

The aim of the present study was to determine the fatty acid composition, the content of *cis*9*trans*11 C18:2 acid (CLA), and lipid quality indices in yogurts made of cow’s milk, available on the Polish market. The test material consisted of: natural yogurts, natural yogurts with additives (muesli, cereal grains), bio yogurts, bio yogurts with additives (millet groats, quinoa, chestnuts), probiotic yogurts, and eco yogurts. All the products were bought in the period from May to June 2021. The conducted research showed that the analyzed yoghurts were characterized by a varying content of fatty acid groups, different values of the calculated lipid quality indices, as well as a different content of conjugated linoleic acid *cis*9*trans*11 C18: 2 (CLA). Natural yogurts with additives had the highest content of polyunsaturated fatty acids (PUFAs) and *n*-3 PUFAs. Natural and bio yogurts with additives had a higher content of *n*-6 PUF than the other analyzed yogurts. The *n*-6/*n*-3 ratio was lower in bio yogurts and eco yogurts. Natural yogurts with additives featured the lowest index of atherogenicity (AI) and index of thrombogenicity (TI) and the highest hypocholesterolemic/hypercholesterolemic ratio (H/H). The fat extracted from the bio yogurts had the highest (0.90% of total fatty acids) mean content of *cis*9*trans*11 C18:2 (CLA). In fat of the other analyzed yogurts, mean CLA content in total content of fatty acids varied from 0.48% in natural yogurts with additives to 0.81% in bio yogurts with additives.

## 1. Introduction

The most popular fermented milk product, yogurt, is a coagulated dairy gel usually produced from cow’s milk through lactic acid fermentation using *Streptococcus salivarius* subs. *Thermophilus* and *Lactobacillus delbruecki* subs. *bulgaricus,* containing at least 10^7^ live bacteria per gram of wet weight. According to the International Dairy Federation, the use of other bacteria, such as *Bifidobacterium* and *Lactobacillus acidophilus*, has been approved to this end as well [1,2]. Due to the growing interest, increase in sales, and implementation of innovative technological solutions, the global dairy products industry is a significant contributor to the economics [3]. In 2019, the value of the global yogurt market was estimated on USD 85.5 billion and it was forecasted that by 2024 it may reach USD 106.6 billion (CAGR of 4.5%) [3,4]. For the European market, the rise in 2021–2026 is estimated at the compound annual growth rate (CAGR) of 3.2% [5]. In Europe, the production of yogurt and other products called acidified milk in the years 2016–2019 was estimated at 8.2 million tons, with Poland contributing 400,000 tons. The average annual yogurt consumption in European households in the last years exceeded 20 kg per capita. For comparison, in Poland it has remained constant at the level of 6 kg since 2014 [2,6].

A short-term positive impact on the retail yogurt market was caused by the outbreak of COVID-19. Consumers were forced to stay at homes and prepare meals by themselves. Moreover, a rise in the online sales of both dairy and non-dairy yogurt products was observed by online grocery delivery platforms during the lockdown period. As a result of the pandemic, consumers aware of the functional health benefits of yogurts were more likely to buy them to follow better-balanced diets [5].

Yogurts are valued for their sensory properties, including a refreshing aroma and a slightly sour taste imparted by lactic acid fermentation. To attract consumers, reduce the acid flavor, and enhance health-promoting effects, many various ingredients are added to the yogurt, including common fruits, fruit seed extracts, vegetables (cucumber, tomato, beet), nuts (walnuts, hazelnuts, almond and pistachios), muesli, and spice oleoresins (cardamon, nutmeg, cinnamon) [7,8]. According to Hlédik and Lógó [9], the most popular among consumers are products containing fruit juices or pulps of strawberry, forest fruit, peach, sour cherry, and raspberry. Yogurts containing rare fruit species, such as species with geographically limited availability, are more frequently available on the dairy market. The examples include products supplemented with pomegranate and jacaranda seeds, mulberry fruit, or goji berries. Moreover, yogurts with added fish oil, fiber, resistant starch, lutein, plant sterols, green tea, and green coffee are available [7,8,10,11]. Moreover, manufacturers compete in adding various combinations of lactic acid bacteria (LAB) with probiotic effects. The listed supplements not only improve the sensory and functional properties by modifying taste, color, and texture, but also provide bioactive phytochemicals, enzymes, and antioxidants as well as change the typical microbiological features [8]. A growing segment of the yogurt market includes bio and eco products containing organic ingredients.

The observed rise in health consciousness among the Europeans favors the growth of the yogurt market [7,10]. The health benefits of yoghurt and other sour milk products have been largely investigated due to their positive effect on the intestinal flora [12]. The numerous health benefits include, among others, improved lactose digestion, protection against gastrointestinal distress, enhanced immune function together with a reduced risk of many diseases, including civilization ones, e.g., obesity and cancers. The role in decreasing atopic diseases, lowering blood cholesterol, enhancing short chain fatty acid production, and increasing protein and calcium assimilation should also be emphasized [7,8,11,13,14,15,16]. These mentioned benefits of yogurt consumption are largely due to the presence of probiotics, live and active bacterial cultures that promote gut health. According to the FAO/WHO, probiotics are defined as live microbes which when administrated in adequate amounts are beneficial to the host in many ways [17]. The ability of probiotic bacteria, mostly those belonging to the genera *Lactobacillus* and *Bifidobacterium*, to synthesize vitamins (such as vitamin K) and most of the water-soluble B vitamins (biotin, cobalamin, folates, nicotinic acid, panthotenic acid, pyridoxine, riboflavin, and thiamine) in humans deserves special attention [18,19]. Besides the ability to produce vitamins, probiotic bacteria are also potent to synthesize other valuable compounds, such as conjugated linoleic acid (CLA) [20,21,22].

The term conjugated linoleic acid (CLA) refers to a group of isomers of linoleic acid (C18:2) in which occur a conjugated system of double bonds. Among these isomers, the most important are: *cis*9*trans*11 (also known as rumenic acid, RA) and *trans*10*cis*12. [23]. CLA is an intermediate in the rumen hydrogenation of linoleic acid, whereas *trans* vaccenic acid VA (C18:1 *trans*11) is a common intermediate in the bio-hydrogenation of linoleic and α-linoleic acids [24]. According to Griinari et al. [25], the endogenous synthesis of CLA from *trans* vaccenic acid represents the primary source of CLA in milk fat. The *cis*9*trans*11 C18:2 is the main CLA component, accounting for 72% to over 90% of the total CLA in ruminant fat [20,21,22,26,27]. The *cis*9*trans*11 C18:2 (CLA) displays a number of health-positive properties [28,29,30,31,32]. Many factors can determine the content of this acid in milk fat, e.g., animal nutrition, breed, age as well as lactation period [24,26,33,34,35,36,37]. Beside milk quality factors, the technological process (i.e., heat treatment of milk, additives, starter cultures, ripening period and storage temperature) affects the quality of fermented dairy products, including their fatty acid profile [38,39,40,41,42,43,44,45,46,47,48]. Dairy products are the main natural source of CLA in the human diet.

Given that the fatty acid profile of milk and dairy products is an important factor affecting their nutritional value, the aim of this study was to determine the fatty acid composition, the content of *cis*9*trans*11 C18:2 (CLA) and lipid quality indices in yogurts available on a daily basis to consumers in retail sale.

## 2. Materials and Methods

### 2.1. Samples

The samples represented different and the most popular groups of yogurts, including products labeled as natural (10 samples), bio yogurts (4 samples), probiotic yogurts (4 samples), eco yogurts (6 samples), and natural and bio yogurts with various additives: natural yogurts with muesli, cereal grains (6 samples), and bio yogurts with millet groats, quinoa, chestnuts) (6 samples). Only the labels of bio yogurts indicated which strains of yogurt bacteria were added in the production process. Most often, on the product label it was stated that the product contains *Lactobacillus acidophilus* and *Bifidobacterium lactis*. Labels of the remaining products provided only information that they contained live yogurt bacteria. The analyzed products were from different producers. All were bought on the Polish market between May and June 2021. All samples were analyzed in duplicate.

### 2.2. Fatty Acid Composition

#### 2.2.1. Fat Extraction

The Folch method was used to extract the fat from analyzed yogurts [49]. For this purpose, 10 ± 0.01 g of sample was homogenized (IKA Ul-tra-Turrax^®^T18 digital) for 1 min with 100 mL of methanol. The next step was to add 100 mL of chloroform and the mixture was subject to homogenization for 2 min. The prepared mixture was then filtered into a 500 mL glass cylinder. Hence, 200 mL chloroform: methanol (2:1 *v/v*) was added and mixed with the solid residue, homogenized again for 3 min, and then transferred to the same cylinder. To the total filtrate, 0.88% sodium chloride in water was added (in the amount constituting 1/4 volume of filtrate), shaken vigorously for 1 min, and left overnight to allow the separation of the layers. Next, a water pump was used to remove the upper layer and the lower layer was washed twice with a water–methanol mixture (1:1 *v/v*), then filtered through anhydrous (VI) sodium sulfate. The solvent was evaporated. The separated fat was used to prepare the methyl esters.

#### 2.2.2. Preparation of Fatty Acid Methyl Esters

The IDF method (ISO 15884:2002) was applied to prepare the fatty acid methyl esters [50]. N-hexane and 2M KOH in methanol were added to the fat sample. The mixture prepared in this way was vigorously shaken. Then, the sodium hydrogen sulphate (NaHSO_4_ × H_2_O) was added, and the mixture was centrifuged for 3 min (1000 spins/minute). The methyl esters prepared in this way were determined by gas chromatography method.

#### 2.2.3. Gas Chromatography (GC) Analysis

Chromatographic separation was performed using Hewlett Packard 6890 gas chromatograph (Műnster, Germany) with a flame ionization detector (FID) and a 100 m capillary column (produced by Chrompack, Middelburg, The Netherlands) with CP Sil 88 phase. The column diameter was 0.25 mm, the film was 0.20 μm thick. Sample injection volume was 0.4 μL (split: 50:1). The conditions of separation were as follows: carrier gas, helium, 1.5 mL/min flow rate; column temperature, −60 °C, 5 °C/min increase to 180 °C; detector temperature −250 °C; injection temperatures 225 °C.

#### 2.2.4. Identification and Calculation of Fatty Acids

Methyl esters of fatty acids were identified according to their retention times, after comparison with retention times of methyl esters of the reference milk fat fatty acids (BCR Reference Materials) of the CRM 164 symbol, and literature data [51,52,53,54]. The *cis*9*trans*11 C18:2 (CLA) isomer was identified using a mixture of CLA methyl esters (Sigma-Aldrich, Germany). To calculate the percentage of fatty acids, the proportions of the peak areas of individual acids in relation to the total area of all identified peaks were used.

#### 2.2.5. The Lipid Quality Indices

A method by Medeiros et al. was used for the calculation of hypocholesterolemic fatty acids (DFA) [55]:

DFA = UFA + C18:0

Hypercholesterolaemic fatty acids (OFA)

OFA = C12:0 + C14:0 + C16:0

The index of atherogenicity (AI) and index of thrombogenicity (TI) indices were calculated using the following formulae according to Ulbricht and Southgate [56] and Osmari et al. [57]:

AI = (C12:0 + (4 × C14:0) + C16:0)/(*n*-3 PUFA + *n*-6 PUFA + MUFA)

TI = (C14:0 + C16:0 + C18:0)/((0.5 × C18:1) + (0.5 × sum of other MUFA) +

0.5 × *n*-6 PUFA) + (3 × *n*-3 PUFA) + *n*-3 PUFA/*n*-6 PUFA))

Hypocholesterolaemic/hypercholesterolemic ratio (H/H) was calculated according to Ivanova and Hadzhinikolova [58]:

H/H = (C18:1*n*-9 + C18:2*n*-6 + C18:3*n*-3)/(C12:0 + C14:0 + C16:0)

### 2.3. Statistical Analysis

Significant differences (*p* < 0.05) in the content of fatty acids, CLA, and lipid quality indexes in analyzed yogurts were estimated using a one-way analysis of variance (ANOVA) with the Duncan’s test. The STATISTICA ver. 13.1 software (Statsoft, Kraków, Poland) was used [59].

## 3. Results and Discussion

### 3.1. Fatty Acid Composition

Milk fat comprises approximately 400 different fatty acids (FAs) of various chain lengths, most of which are saturated fatty acids (SFAs), with a lesser amount of monounsaturated fatty acids (MUFAs) and polyunsaturated fatty acids (PUFAs). This diversity of FAs makes its composition very complex [23,60,61]. The fat of cow’s milk contains about 70% of SFAs, 25% of MUFAs, and 2–5% PUFAs [62]. Short-chain fatty acids (SCFAs, C4:0–C10:0), which are an important factor in the promotion of human health, represent approximately 10% of all saturated fatty acids [61,63]. Fat extracted from the analyzed yogurts was characterized by diversified contents of fatty acids (Table 1). In all analyzed products saturated fatty acids (SFAs) were found to prevail. The significantly (*p* < 0.05) lowest SFAs content (54.81% of total fatty acids) was determined in fat from natural yogurts with additives compared to fat from the other analyzed yogurts. The fat from natural yogurts with additives and probiotic yogurts had the lowest content of SCFAs (8.28% and 8.67% of total fatty acids, respectively) (Table 1). Fat from the other analyzed yogurts was characterized by a significantly higher (*p* < 0.05) content of these acids. Considering the fact that these acids play important biological functions, these yoghurts can better support the healthy functioning of the body. The content of MUFAs was the highest in fat from the tested probiotic yogurts (27.99% of total fatty acids), while the lowest was in fat extracted from bio yogurt and bio yogurts with additives (25.24 and 25.07% of total fatty acids, respectively). The highest content of PUFAs (7.89% of total fatty acids) was determined in fat from natural yogurts with muesli and cereal grains. Significantly lower (*p* < 0.05) contents of PUFAs were found in other analyzed yoghurts (Table 1). The higher content of SCFAs (from 10.0% to 11.33% of the total fatty acids) in natural yoghurts available on the Polish market was found in previous study by Paszczyk and Rafałowski [64]. The SFAs in the yoghurts studied by these authors ranged from 59.66% to 63.14%. The mean contents of MUFAs accounted for 25.37%, and that of PUFAs for 3.12%. Differences in the fatty acid composition of yogurts may result both from differences in the composition of raw milk and differences in the production technology. The fatty acid profile in dairy products may be influenced by quite a number of already known intrinsic factors, such as stage of lactation, breed, or genotype, or extrinsic factors, e.g., nutrition, season, dairy production system, and feeding ration [33,36,37]. All previously listed factors can vary greatly among the countries, and therefore the same product produced in a different country can vary in terms of composition. The concentrations and the profile of fatty acids in the final products are primarily dependent on the fatty acid contents of raw milk. Furthermore, the impact of dairy processing technological stages, such as heat treatment, homogenization, fermentation, and storage, on fatty acids profile should also be considered. Little research has been done in this area and the obtained data vary. According to the research by Khan et al. [65], high milk temperature caused an increase in short chain fatty acids (SCFAs), medium chain fatty acids (MCFAs) and a decrease in long chain fatty acids (LCFAs) after pasteurization and boiling, while the studies of Pestana et al. [66] indicate a decrease in SCFAs. According to Santos Júnior et al. [67], after pasteurization, milk contained more SCFAs and PUFAs and less MUFAs. No significant differences were found in pasteurized and UHT milk samples in SCFAs, SFAs, MUFAs, and PUFAs contents in the previous study of Paszczyk and Łuczyńska [68].

The fat content and fatty acid profile, as well as the content of protein, vitamins, and macroelements are important qualities of yoghurt [69,70]. The composition of the milk used for yoghurt production has an impact on its nutrient composition as well as many other factors, including genetic and individual mammalian differences, feed, stage of lactation, age, and environmental factors, such as season of the year [24,36,37]. Not only milk processing (temperature, exposure to light, time of heating, storage conditions), but also the process of lactic acid fermentation and the changes in milk constituents that come as the result of this process can have an effect on the nutritional value of the final product. Various additives added to the yogurt can significantly improve the health and sensory properties of milk fermented products. Finally, yogurt nutritional composition and quality can vary due to the source and type of milk solids added before fermentation, species and strains of bacteria used, as well as time and temperature of the fermentation process [71,72,73].

The present study indicates that fat extracted from yogurts containing muesli and cereal grains was characterized by a significantly higher (*p* < 0.05) content of *n*-3 and *n*-6 PUFAs (Table 1) compared to the other tested products. The *n*-6/*n*-3 ratio ranged from 1.83 in fat from bio yogurts to 5.07 in fat from natural yogurts. In the study conducted by Månsson [61], the *n*-6/*n*-3 PUFAs ratio in milk fat was 2.3, whereas in fat from yogurts analyzed by Paszczyk and Łuczyńska [68] it was 4.77 in natural yogurts, 3.04 in bio-yogurts, and up to 10.59 in fat from yogurts with fruit and cereal grains.

The determination of the contents of *n*-6 and *n*-3 fatty acids is especially important since their adequate intake is essential for health [74]. Those fatty acids are linked with a reduced risk of many diseases such as cardiovascular, type two diabetes, hypertension, cancer, and certain disruptive neurological functions [75,76,77,78,79,80,81]. Solutions that will lead to an increased intake of these functional food components are needed for better human nutrition. An *n*-6 to *n*-3 ratio of 20–30:1 is typical for western diets, while the ratio of 4:1 or less is thought to be ideal. Diets that include excessive amounts of *n*-6 PUFAs and a high *n*-6/*n*-3 ratio promote the pathogenesis of many diseases, whereas increased levels of *n*-3 PUFAs (a lower *n*-6/*n*-3 ratio) exert suppressive effects [80]. In our study, bio yogurts together with eco yogurts had the lowest *n*-6/*n*-3 ratio and thus their FA composition indicated that they may best support healthy functioning of the body.

### 3.2. The Content of CLA

**Figure 1:**
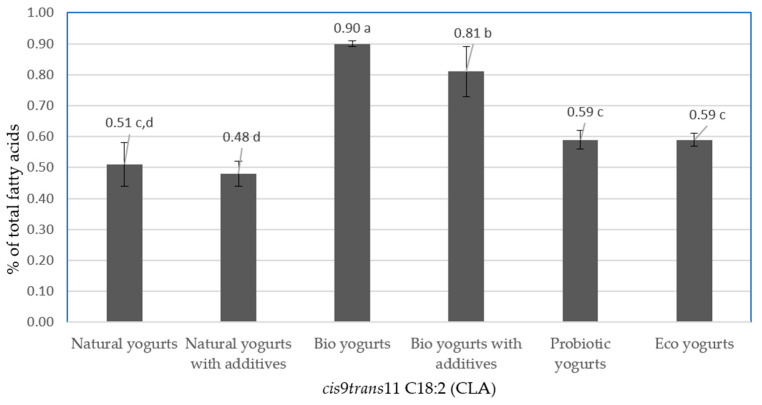
Mean content of *cis*9*trans*11 C18:2 (CLA) in fat from the analyzed yogurts (% of total fatty acids). a, b, c, d—values with different letters differ significantly (*p* < 0.05).

Figure 1 presents the contents of *cis*9*trans*11 C18:2 acid (CLA) in fat from the analyzed yogurts. The data indicate that the tested products were characterized by varied contents of CLA. Fat from bio yogurts had the highest mean CLA content (0.70% of total fatty acids). Significantly lower mean CLA contents (*p* < 0.05) were found in fat from the other analyzed products. The lowest content of CLA (0.48% of total fatty acids) was found in natural yogurts with additives (muesli, cereal grains). According to Żegarska et al. [82], the CLA content of milk fat ranged from 1.06% to 1.76% in pasture period and from 0.32% to 0.52% in winter season. The CLA content in yogurts analyzed by Żegarska et al. [83], purchased from January to February, ranged from 0.37% to 0.49% of total fatty acids, while in the yogurts bought from June to July, from 0.97% to 1.25%. In commercial yogurts tested by Paszczyk et al. [84], which were manufactured from January to March, the mean CLA contents in natural yogurts and bio yogurts were at the comparable levels of 42% and 43%, respectively. Paszczyk and Rafałowski [64] found similar contents of CLA in fat of natural yogurts purchased between February and April in retail stores.

### 3.3. The Lipid Quality Indices

The values of lipid quality indices, DFA (desirable hypocholesterolemic fatty acids), OFA (hypercholesterolemic fatty acids), TI (the thrombogenicity index), and AI (the atherogenicity index), varied in the analyzed yogurts (Table 2). The content of DFAs, which is the sum of unsaturated fatty acids (UFAs) and C18:0 (stearic acid), was higher in fat from eco yogurts, probiotic yogurts and natural yogurts with additives, while being significantly lower (*p* < 0.05) in the other tested products. Fat from natural yogurts, bio yogurts, and bio yogurts with additives had significantly higher (*p* < 0.05) contents of OFAs than the others analyzed yogurts. The OFAs content is influenced by the content of saturated fatty acids such as C12:0, lauric acid, C14:0, myristic acid, and C16:0, palmitic acid. The values of AI were significantly higher (*p* < 0.05) in fat from natural yogurts, bio yogurts, and bio yogurts with additives compared to the other analyzed yogurts. The lowest value of TI (1.68) was determined in fat from natural yogurts with additives (Table 2). Fat from the other analyzed yogurts had significantly higher (*p* < 0.05) values of TI. The AI and TI indices take into account the different effects that single fatty acids might have on human health. They are related to the risk of development of cardiovascular diseases [56]. According to Ivanova and Hadzhinikolova [58], the higher values of these coefficients, the higher the risk of developing cardiovascular diseases, as AI indicates the risk of diseases such as atherosclerosis (deposition of fat in the walls of the arteries) and TI determines the possibility of blood clots. Thus, in our study, the natural yogurts with additives, such as muesli and cereal grains, showed the highest protective potential against the mentioned health problems.

The hypocholesterolemic/hypercholesterolemic (H/H) ratio is related to the functional activity of fatty acids in the metabolism of lipoproteins for plasma cholesterol transport and to the risk of cardiovascular disease. According to Santos-Silva et al. [85], higher values of this indicator are more desirable. In the current study, the highest H/H ratio was found in fat from natural yogurts with additives (0.70) (Table 2). In contrast, fat from the other analyzed yogurts, including bio yogurts (0.41), had significantly lower (*p* < 0.05) H/H values. Similarly, in the previous study [68], the highest H/H index was observed in natural yogurts with additives (0.70), whereas it was significantly lower in natural and bio yogurts, reaching 0.55 and 0.54, respectively. It can be concluded that the introduction of selected additives to yogurt can significantly affect the level of H/H.

## 4. Conclusions

Among all fermented milk products, yoghurts are the most popular choice of consumers all over the world. Increasingly aware consumers want to choose high-quality products with health-promoting properties and a high sensory value. The conducted study provides basic knowledge on the contents of fatty acids, content of *cis*9*trans*11 C18:2 (CLA), and the lipid quality indices in market yogurts. The results showed that yogurts of different categories, i.e., natural, bio, probiotic, eco, and with additives, have different contents of PUFAs and *n*-3 PUFAs, *n*-6/*n*-3 ratio, as well as values of AI and TI indices and the H/H ratio. This information is important for consumers when planning a rational diet, but also for producers who are engaged in a continuous search for innovative products.

Nevertheless, our research shows that on the basis of the evaluated lipid quality indices, the consumer can choose, from the yoghurt offered in retail, products with more beneficial pro-health effects. Examples are bio yogurts together with eco yogurts with the lowest *n*-6/*n*-3 ratio as well as natural yogurts with additives with the lowest AI and TI levels.

## Figures and Tables

**Table 1:** Fatty acid composition (% of total fatty acids) in fat from the analyzed yogurts.

		Natural Yogurts	Natural Yogurts with Additives (Muesli, Cereal Grains)	Bio Yogurts	Bio Yogurts with Additives (Millet Groats, Quinoa, Chestnuts)	Probiotic Yogurts	Eco Yogurts
n		10	6	4	6	4	6
**ΣSCFAs**	**Mean**	**9.32 ^a^**	**8.28 ^b^**	**9.30 ^a^**	**9.33 ^a^**	**8.67 ^b^**	**9.60 ^a^**
	SD	0.83	0.47	0.03	0.17	0.03	0.17
	Min–Max	8.06–10.08	7.43–8.80	9.29–9.33	8.98–9.49	8.64–8.70	9.41–9.79
**ΣSFAs**	**Mean**	**58.94 ^a^**	**54.81 ^b^**	**58.35 ^a^**	**58.60 ^a^**	**57.97 ^a^**	**57.41 ^a^**
	SD	0.63	1.17	0.13	1.03	0.23	0.05
	Min–Max	58.16–59.98	53.39–57.96	58.22–58.52	57.92–60.92	57.74–58.26	57.36–57.46
**ΣMUFAs**	**Mean**	**26.96 ^a,b^**	**26.00 ^b,c^**	**25.24 ^c^**	**25.07 c**	**27.99 ^a^**	**27.85 ^a,b^**
	SD	1.33	1.15	1.04	0.59	0.06	0.28
	Min–Max	25.91–30.55	24.89–27.53	23.87–26.31	24.45–26.02	27.92–28.07	27.16–28.16
**ΣPUFAs**	**Mean**	**3.32 ^c^**	**7.89 ^a^**	**3.95 ^b,c^**	**4.96 ^b^**	**2.85 ^c^**	**3.36 ^b,c^**
	SD	0.25	2.64	0.04	0.74	0.41	0.04
	Min–Max	3.14–3.94	5.09–11.03	3.90–4.00	3.85–5.79	2.42–3.39	3.32–3.40
***n*-3****	**Mean**	**0.37 ^b^**	**3.46 ^a^**	**0.97 ^a^**	**0.93 ^a^**	**0.39 ^a^**	**0.69 ^a^**
	SD	0.06	2.84	0.01	0.11	0.01	0.01
	Min–Max	0.29–0.43	0.43–6.76	0.78–0.81	0.79–1.07	0.38–0.39	0.67–0.70
***n*-6****	**Mean**	**1.82 ^c^**	**3.47 ^a^**	**1.46 ^c^**	**2.74 ^b^**	**1.34 ^c^**	**1.41 ^c^**
	SD	0.21	0.30	0.01	0.58	0.43	0.01
	Min–Max	1.61–2.08	3.15–3.81	1.45–1.47	1.59–3.07	0.91–1.91	1.40–1.42
***n*-6/*n*-3**	**Mean**	**5.07 ^a^**	**3.32 ^a,b^**	**1.83 ^c^**	**2.64 ^c^**	**3.43 ^a,b^**	**2.06 ^c^**
	SD	1.44	3.97	0.01	0.44	1.10	0.03
	Min–Max	3.82–7.26	0.48–7.84	1.82–1.85	1.93–3.04	2.30–4.87	2.02–2.09

n—number of samples; Mean—mean value; SD—standard deviation; Min—minimum value; Max—maximum value; ^a,b,c^—values in rows with different letters differ significantly (*p* < 0.05); ΣSCFAs—sum of short-chain fatty acids (C4:0–C10:0); ΣSFAs—sum of medium- and long-chain saturated fatty acids; ΣMUFAs—sum of monounsaturated fatty acids; ΣPUFAs—sum of polyunsaturated fatty acids.

**Table 2:** Nutritional indices in the analyzed yogurts.

		Natural Yogurts	Natural Yogurts with Additives (Muesli, Cereal Grains)	Bio Yogurts	Bio Yogurts With Additives (Millet Groats, Quinoa, Chestnuts)	Probiotic Yogurts	Eco Yogurts
n		10	6	4	6	4	6
**DFAs**	**Mean**	**39.63 ^b^**	**42.71 ^a^**	**38.78 ^b^**	**39.35 ^b^**	**41.89 ^a^**	**42.78 ^a^**
	SD	1.74	2.28	1.12	1.69	0.50	0.44
	Min–Max	37.70–44.01	39.12–44.66	37.31–39.92	36.97–41.53	41.37–42.54	42.27–43.28
**OFAs**	**Mean**	**49.59 ^a^**	**46.00 ^b^**	**48.77 ^a^**	**49.29 ^a^**	**46.92 ^b^**	**45.85 ^b^**
	SD	0.85	1.58	0.16	1.43	0.20	0.16
	Min–Max	48.47–51.13	44.57–49.02	48.61–48.98	47.68–52.21	46.71–47.19	45.67–46.02
**AI**	**Mean**	**2.73 ^a^**	**2.04 ^c^**	**2.86 ^a^**	**2.81 ^a^**	**2.50 ^b^**	**2.40 ^b^**
	SD	0.15	0.18	0.12	0.17	0.04	0.05
	Min–Max	2.36–2.88	1.70–2.23	2.74–3.02	2.64–3.10	2.45–2.54	2.34–2.45
**TI**	**Mean**	**3.19 ^a^**	**1.68 ^b^**	**3.10 ^a^**	**3.00 ^a^**	**3.14 ^a^**	**2.90 ^a^**
	SD	0.33	0.58	0.11	0.18	0.03	0.01
	Min–Max	2.29–3.43	1.28–2.77	2.98–3.25	2.84–3.28	3.10–3.16	2.89–2.91
**H/H**	**Mean**	**0.44 ^b^**	**0.70 ^a^**	**0.41 ^b^**	**0.42 ^b^**	**0.45 ^b^**	**0.47 ^b^**
	SD	0.04	0.24	0.01	0.03	0.01	0.01
	Min–Max	0.41–0.55	0.54–1.17	0.41–0.42	0.38–0.45	0.44–0.46	0.46–0.47

n—number of samples; Mean—mean value; SD—standard deviation; Min—minimum value; Max—maximum value; ^a,b,c^—values in rows with different letters differ significantly (*p* < 0.05); DFA—hypocholesterolemic fatty acids (ΣUFA + C18:0); OFA—hypercholesterolemic fatty acids (ΣSFA-C18:0); AI—Index of Atherogenicity; TI—Index of Thrombogenicity; H/H—hypocholesterolemic/hypercholesterolemic ratio.

## Data Availability

Data is contained within the article.
